# Handshake antimicrobial stewardship for adult surgical patients

**DOI:** 10.1017/ash.2024.498

**Published:** 2025-02-12

**Authors:** Abby Kosharek, Elizabeth Neuner, Emily Welch, Spenser January, Alice Bewley, Kevin Hsueh, Sena Sayood

**Affiliations:** 1 Department of Pharmacy, Barnes-Jewish Hospital, St. Louis, MO, USA; 2 Division of Infectious Diseases, Washington University School of Medicine in St. Louis, St. Louis, MO, USA

## Abstract

**Objective:**

Evaluate the effects of handshake stewardship on adult general surgical units.

**Design:**

Retrospective quasi-experimental pre- and post-intervention study.

**Setting:**

A total of 1,278 bed academic medical center with a level 1 trauma center in St. Louis, Missouri.

**Patients:**

Adults admitted to general surgery units.

**Intervention:**

Once weekly handshake antimicrobial stewardship rounds were initiated in January 2022 on adult general surgery units. The handshake stewardship team consisted of an infectious diseases (ID) physician and pharmacist who reviewed charts of patients receiving systemic antimicrobials without a formal ID consult. Antimicrobial recommendations were delivered in person to general surgery teams including trauma, geriatric trauma, and emergency/general surgery.

**Results:**

A total of 1,241 charts were reviewed during the post-implementation period with 391 interventions. Seventy-two percent of those interventions were accepted and the acceptance rate improved over the 18-month post-implementation period. Total antimicrobial usage significantly decreased between the pre- and post-implementation period (608 vs 542 d of therapy/1,000 d present, *P* = 0.004). An interrupted time series found that there was an immediate (*P* < 0.001) and sustained (*P* < 0.001) decrease in antibiotic spectrum index during the post-implementation period. No difference was found for in-hospital mortality between the pre- and post-implementation periods [28 (1%) vs 29 (1%), *P* = 0.791].

**Conclusion:**

A once-weekly handshake antimicrobial stewardship program was successfully implemented in general surgery units. Antimicrobial use significantly decreased without negatively impacting hospital mortality.

## Introduction

Handshake stewardship is a unique approach to antimicrobial stewardship and is considered a leading practice by The Joint Commission.^1^ Handshake stewardship is a form of prospective audit and feedback consisting of the collaborative review of all antimicrobial orders by an infectious diseases (ID) physician and ID pharmacist followed by in-person rounding with primary teams to communicate recommendations.^1,2^ Most descriptions of this intervention have been in pediatric patient populations and adult intensive care units (ICU).^3–6^ These previous studies have shown implementing handshake stewardship in these populations reduced overall antimicrobial use without unfavorable patient outcomes.^3–6^ Descriptions of handshake stewardship interventions in other specific subpopulations (eg patients with hematologic malignancies) are limited.^7^ In particular, data specific to adult surgery patients is lacking. In January 2022, the existing handshake stewardship service at Barnes-Jewish Hospital was expanded to include patients on surgical floor units. This study evaluated the effect of handshake stewardship on antimicrobial use within the adult general surgery population.

## Methods

Barnes-Jewish Hospital is a tertiary care academic medical center with a level 1 trauma center located in St. Louis, Missouri. Antimicrobial stewardship activities at the hospital include prior authorization, 72-hour prospective audit and feedback of certain antimicrobials, institution-specific guidelines for select infectious diseases, and clinical decision support within the electronic health record (EHR) (see supplement for additional details). Additionally, a handshake antimicrobial stewardship program was initiated as a quality improvement activity in August 2021 for thirteen medicine units with rounds occurring three times weekly.^8^

Beginning in January 2022, the handshake stewardship service was expanded to the adult surgical units. This study focuses specifically on the two general surgery units (61 beds) including patients cared for by the trauma, geriatric trauma, and acute and critical care surgery teams. Once weekly, the handshake stewardship team reviewed patients receiving antimicrobials admitted to adult surgery units. Reviews were divided between an ID physician and ID pharmacist (additional workflow details in supplement). The team rounded in person to discuss recommendations with the physician associates and nurse practitioners responsible for floor management of surgical patients. Following in-person rounds, recommendations were documented in the pharmacy intervention system within the EHR (Epic Systems Corporation, Verona WI).

Inclusion criteria were all patients admitted to one of the two general surgery units who received antibiotics during the eighteen months pre- (July 2020-December 2021) and post- (January 2022-June 2023) intervention. No patients who received antibiotics were excluded.

The primary outcome was antimicrobial use measured in days of therapy (DOT) per 1,000 days present (DP). DOT/1,000 DP was obtained from the Centers for Disease Control and Prevention’s National Healthcare Safety Network (NHSN) Antimicrobial Use module. Baseline demographics and secondary outcome variables were obtained from the EHR. Intravenous and oral antibiotic usage was captured by assessing digestive DOT (dDOT) over total DOT (tDOT).^9^ To examine the impact of this intervention on a metric that incorporates antimicrobial spectrum of drug exposure, the antibiotic spectrum index (ASI) was calculated using an expanded and modified version of Gerber et al.’s (supplementary appendix).^10,11^ Lastly, patient safety outcomes included in-hospital mortality, hospital, and ICU length of stay, 30-day readmission, and hospital-onset *Clostridioides difficile* infection as per the NHSN criteria.

This study was reviewed and approved by the local institutional review board. Antimicrobial interventions were analyzed using descriptive statistics. For comparisons of pre- and post-implementation groups, categorical data was compared via Chi-squared or Fisher’s exact test, and continuous data was compared via Mann-Whitney U or Student’s t-test. An interrupted time series analysis comparing both the immediate effect of the intervention and change in the slope in DOT/1,000 DP of the pre- and post-implementation period was performed. Models were checked for seasonality via the autocorrelation function and autocorrelation was not detected. A prespecified three-month lag time was applied to the interrupted time series to evaluate for a delayed intervention effect. All data were analyzed using SPSS Statistics version 25 (IBM Corp, Armonk, NY) and R version 4.3.3 (R Foundation for Statistical Computing, Vienna, Austria).

## Results

In total 2,340 patients were included in the pre-implementation period and 2,247 patients were included in the post-implementation period. Age significantly differed between the pre-implementation and post-implementation groups (60 vs 63 years, *P* < 0.001). There was also a significant difference in the type of surgical procedure performed between the pre- and post-implementation group for the subcategory of general surgery 46% vs 40%, *P* = 0.010. All other baseline characteristics were similar between the groups (Table 1).

**Table 1.** Baseline characteristics

Baseline Characteristics	Pre-Implementation	Post-Implementation
	Jul 2020 – Dec 2021	Jan 2022 – June 2023
	(N = 2,340)	(N = 2,247)
Age, years, median (IQR)	60 (40–70)	63 (45–75)
Sex, male, n (%)	1,247 (53)	1,158 (52)
Race, n (%)		
Caucasian	1,516 (65)	1,466 (65)
Black	753 (32)	694 (31)
Other	71 (3)	85 (4)
Charlson comorbidity index, median (IQR)	1 (0–3)	1 (0–3)
Penicillin allergy, n (%)	216 (9)	209 (9)
Cephalosporin allergy, n (%)	22 (1)	31 (1)
ANC < 500 cells/µL, n (%)	7 (0.3)	4 (0.2)
Solid organ transplant, n (%)	40 (2)	51 (2)
ID consult, n (%)	302 (13)	213 (10)
Number of surgical procedures, median (IQR)	1 (1–2)	1 (1–2)
No surgery performed during admission, n (%)	518 (22)	480 (21)
Surgery case class, n (%)	n = 1,822	n = 1,769
Emergent	1,607 (69)	1,607 (71)
Elective	215 (9)	162 (8)
Type of primary surgical procedure, n (%)	n = 1,822	n = 1,769
Orthopedics	841 (46)	866 (49)
General	830 (46)*	716 (40)*
Other**	151 (8)	185 (10)

*General surgery procedure type significantly differed from both groups

**Other included cardiovascular, cardiothoracic, neurosurgery, ophthalmology, oral/maxillofacial, otolaryngology, plastics, urology, OBGYN, and podiatry

**Figure 1.**
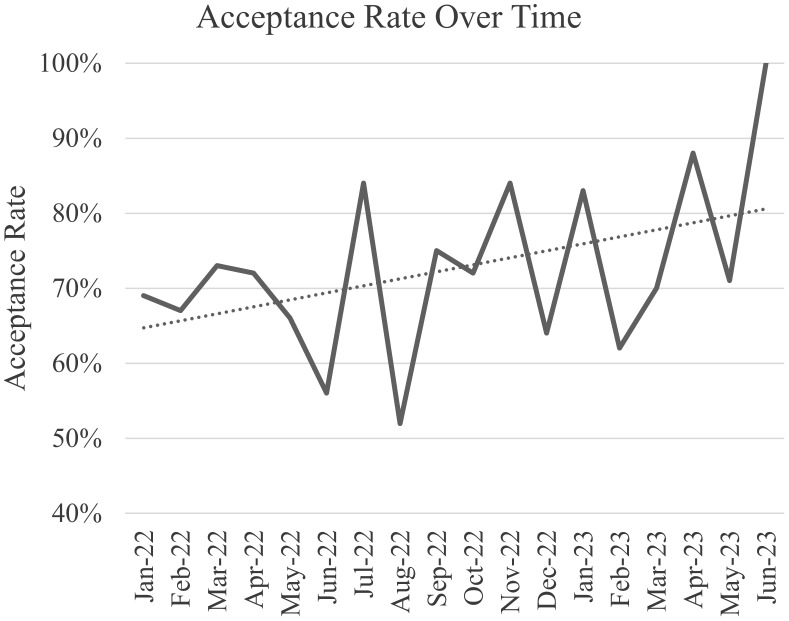
Acceptance rate over time Acceptance rate of antimicrobial interventions during post-implementation period by month.

In the post-intervention period, the handshake antimicrobial stewardship team reviewed 1,241 patient charts, rounded 70 days, and made 391 recommendations (Table 2). The three most common recommendations were discontinuation (40%), de-escalation (18%), and broaden coverage/address absent therapy (11%). Overall acceptance rate was 72%, with acceptance rate trending upward during the post-implementation period from 69% in January 2022 to 100% in June 2023 (Figure 1). Acceptance rate varied by intervention type, ranging from 92% for recommendations to broaden coverage/address absent therapy to 44% for recommending ID consult.

**Table 2.** Handshake antimicrobial stewardship interventions

Type of recommendation, n (%)	Total n = 391
Discontinue	156 (40)
De-escalation	71 (18)
Broaden coverage/address absent therapy	43 (11)
Recommended ID consult	32 (8)
Shorten/define duration	30 (8)
Dose optimization	10 (3)
Other*	49 (12)
Antimicrobial intervened upon, n (%)^	Total n = 435
Ceftriaxone	77 (17)
Cefepime	54 (12)
Vancomycin	52 (12)
Metronidazole	26 (6)
Sulfamethoxazole/trimethoprim	21 (5)
Ciprofloxacin or Levofloxacin	20 (5)
Meropenem	15 (3)
Fluconazole	12 (3)
Clindamycin	11 (3)
Cefazolin	11 (3)
Doxycycline or Minocycline	11 (3)
Linezolid	9 (2)
Cephalexin	8 (2)
Piperacillin/tazobactam	8 (2)
Ertapenem	7 (2)
Amoxicillin/clavulanate	6 (1)
Nitrofurantoin	6 (1)
Other^	81 (19)
Acceptance rate of top five interventions, n (%)	
Discontinue	114/156 (73)
De-escalation	52/71 (73)
Broaden coverage/address absent therapy	23/25 (92)
Recommended ID consult	14/32 (44)
Shorten/define duration	18/30 (60)

*Other interventions included: Lengthen duration (n = 5), Route changes intravenous to oral (n = 5), Medication safety (n = 5), Monitoring related (n = 3), and Other (n = 31).

^Other antimicrobials included: Micafungin (n = 5), Amoxicillin (n = 3), Ampicillin/sulbactam (n = 3), Acyclovir/Ganciclovir (n = 3) and Other (67).

^Interventions could apply to more than one antimicrobial.

**Figure 2.**
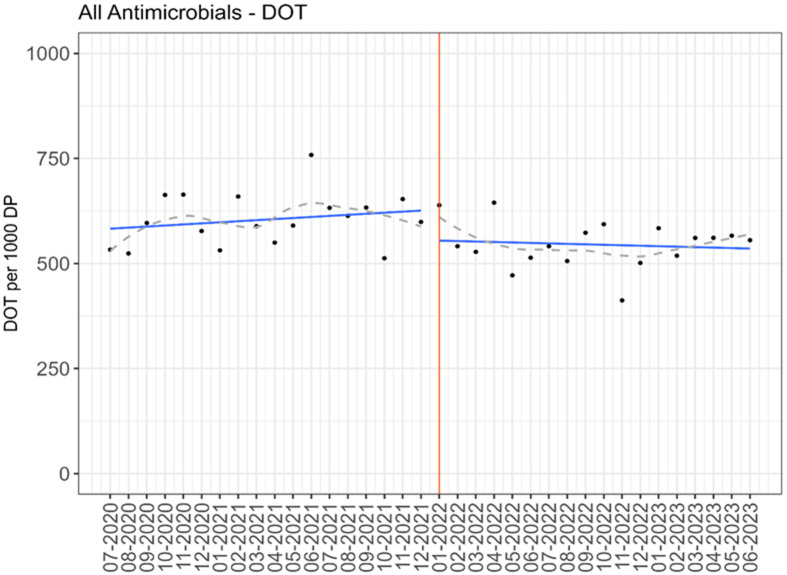
Antimicrobial usage by month on general surgery units (black dots); average consumption from the predicted distribution of the interrupted time series segmented regression model (blue line) with loess smoother (gray dashed line); intervention started at red line.

**Figure 3.**
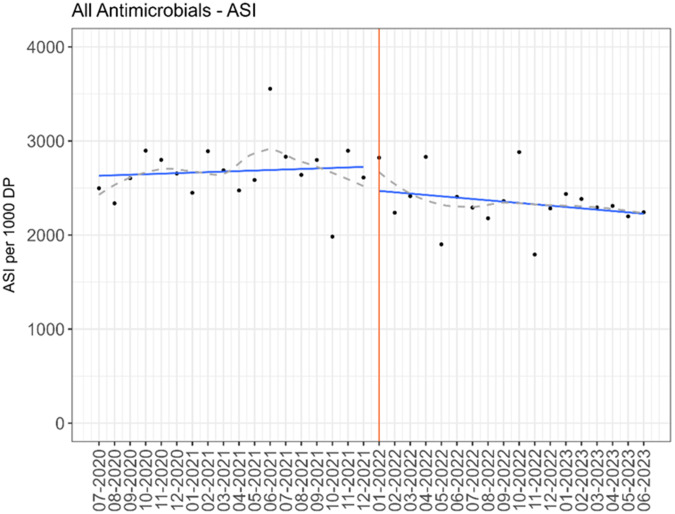
Antimicrobial usage by month on general surgery units (black dots); average spectrum score from the predicted distribution of the interrupted time series segmented regression model (blue line) with loess smoother (gray dashed line); intervention started at red line.

On the general surgery units, there was a significant reduction in antimicrobial usage with an average of 608 pre-implementation compared to 542 DOT/1,000 DP post-implementation of handshake antimicrobial stewardship (*P* = 0.004). The reduction in antimicrobial use was primarily driven by a change in antibacterial usage, with a reduction from 545 to 494 DOT/1,000 DP (*P* = 0.007). Anti-fungal and anti-viral use did not significantly change. All antimicrobial use outcomes are displayed in Table 3. For specific antimicrobial groups and agents, there were several significant differences in use between the pre- and post-implementation periods. There was a significant decrease in aminoglycosides (4 vs 2 DOT/1,000 DP, *P* = 0.014), cephalosporins (197 vs 180 DOT/1,000 DP, *P* = 0.019), and fluoroquinolones (27 vs 21 DOT/1,000 DP, *P* = 0.025). After calculating ASI score from the individual agent DOT/1,000 DP, there was a significant decrease in spectrum score between the pre- and post-implementation time periods (2,839 vs 2,519, *P* = 0.007). While there was an increase in some specific oral agent usage (for example, amoxicillin-clavulanate), there was not a significant increase in overall dDOT/tDOT. The interrupted time series for DOT/1,000 DP for antimicrobial use and ASI are displayed in Figures 2 and 3. There was an immediate decrease in overall antimicrobial use when the intervention was initiated (*P* < 0.001), but the sustained effect did not reach statistical significance (*P* = 0.071). The results of the interrupted time series did not change after applying the prespecified 3-month lag time (supplementary appendix Figure 1). There was an immediate decrease in ASI (*P* < 0.001) and the effect was sustained over the 18-month period (*P* < 0.001). There were similar findings when the 3-month lag time was applied (supplementary appendix Figure 2).

**Table 3.** Antimicrobial use

DOT/1,000 Days Present	Pre-Implementation Jul 2020 – Dec 2021	Post-Implementation Jan 2022 – Jun 2023	P-value
Hospital wide, mean ± SD	755 ± 25	738 ± 24	0.036
General surgery units, mean ± SD			
All antimicrobials	608 ± 69	542 ± 56	0.004
All antibacterials	545 ± 60	494 ± 46	0.007
All antifungals	43 ± 17	36 ± 15	0.21
All antivirals	20 ± 12	12 ± 10	0.05
General surgery units, antimicrobial groups, mean ± SD			
Aminoglycosides	4 ± 2	2 ± 2	0.014
Carbapenemsvvv	51 ± 23	38 ± 15	0.055
Cephalosporins	197 ± 25	180 ± 16	0.019
Fluoroquinolones	27 ± 7	21 ± 9	0.025
MRSA agents	126 ± 23	116 ± 20	0.159
Penicillins and derivatives	54 ± 12	60 ± 15	0.219
Tetracyclines	11 ± 7	15 ± 6	0.115
General surgery units, select antimicrobials, mean ± SD			
1^st^ generation cephalosporins*	72 ± 12	76 ± 10	0.24
2^nd^ generation cephalosporins^	0.03 ± 0.13	0.40 ± 1	0.095
3^rd^ generation cephalosporinsˠ	54 ± 17	48 ± 16	0.262
Cefepime	65 ± 13	55 ± 11	0.014
Meropenem	18 ± 11	12 ± 8	0.061
Ertapenem	31 ± 11	25 ± 9	0.096
Vancomycin	62 ± 12	59 ± 12	0.468
Piperacillin/tazobactam	19 ± 8	21 ± 11	0.742
Amoxicillin/clavulanate	14 ± 6	19 ± 7	0.03
Metronidazole	75 ± 20	65 ± 19	0.126
Remdesivir	0	2 ± 4	0.012
dDOT/tDOT, mean ± SD	0.28 ± 0.05	0.31 ± 0.05	0.072
Antibiotic spectrum index/1,000 days present, mean ± SD	2,839 ± 371	2,519 ± 287	0.007

*1^st^ generation cephalosporins included: cefazolin, cephalexin, and cefadroxil.

^2^nd^ generation cephalosporins included: cefaclor, cefoxitin, cefprozil, cefuroxime, and cefotetan.

ˠ3^rd^ generation cephalosporin included: ceftriaxone, cefdinir, cefixime, cefotaxime, cefpodoxime, and ceftazidime.

In-hospital mortality between study periods was unchanged [28 (1%) vs 29 (1%), *P* = 0.791] (Table 4). Hospital length of stay increased between the pre- and post-implementation period [median 6 d (IQR 4–12) vs 7 d (IQR 4–13), *P* = 0.034], but no difference was found in ICU length of stay [0 (0–1) vs 0 (0–1), *P* = 0.500]. There was no difference in 30-day readmission [315 (14%) vs 300 (13%), *P* = 0.931) and hospital-onset *C. difficile* infection [14 (1%) vs 10 (0.4%), *P* = 0.542] between the pre- and post-implementation group.

**Table 4.** Patient outcomes

Patient Outcomes	Pre-Implementation Jul 2020 – Dec 2021 (n = 2,340)	Post-Implementation Jan 2022 – Jun 2023 (n = 2,247)	P-value
Hospital mortality rate, n (%)	28 (1)	29 (1)	0.791
Length of stay, median (IQR)	6 (4–12)	7 (4–13)	0.034
ICU length of stay, median (IQR)	0 (0–1)	0 (0–1)	0.500
30-day readmission, n (%)	315 (14)	300 (13)	0.931
Hospital-onset *C. difficile* infection, n (%)	14 (1)	10 (0.4)	0.542

## Discussion

The expansion of the handshake antimicrobial stewardship program to adult general surgery units had a measurable impact on antimicrobial use. Overall antimicrobial DOT/1,000 DP significantly decreased, including reductions in specific high-risk agents including aminoglycosides, cefepime, and fluoroquinolones. To our knowledge, this is the first description of the implementation and impact of handshake antimicrobial stewardship on adult surgical units.

One process metric for a successful stewardship implementation is acceptance rate of interventions. For this study, the overall acceptance rate of handshake stewardship interventions was 72%. Handshake stewardship intervention acceptance rates vary between patient populations. The acceptance rate on the surgical units seen in our study was slightly lower than previously reported by handshake stewardship interventions in the pediatric population (86%), internal medicine population (80%), and ICUs (78%).^3,8,12^ This is consistent with prior literature describing lower rates of antimicrobial stewardship acceptance overall by surgical services compared to other service lines.^13^ Previous literature compared antimicrobial recommendation acceptance rates between inpatient surgery and medicine teams in a single center and cited hierarchical structure within surgery teams as a possible cause of lower acceptance rate of antimicrobial recommendations.^14^ In our handshake intervention, most recommendations were given to nurse practitioners or physician associates with varying levels of autonomy related to antimicrobial prescribing. However, one promising finding is that the stewardship recommendation acceptance rates in this study increased over the 18-month post-implementation period. We posit this is due to the long-term positive impact of relationship building, development of trust, and mutual understanding of cultural norms, suggesting handshake stewardship efficiency can improve over time.

After implementing a once-weekly handshake stewardship intervention, we found a statistically significant reduction in overall antimicrobial use in adult general surgery units. We also found a significant decrease in hospital-wide antimicrobial use likely due to the concurrent handshake antimicrobial stewardship rounds implemented three times per week for the thirteen medicine units. In similar studies, handshake teams that rounded daily had a more profound effect than those that rounded weekly.^2,3,8,12^ Handshake antimicrobial stewardship is often considered a resource-intensive intervention; however, this study indicates that even a once-weekly implementation can make a significant impact on antimicrobial use in select patient populations. During the study period, surgical prophylaxis protocols did not change, meaning the antimicrobial trends seen in this study were mostly affected by antimicrobial use outside of prophylaxis. Despite seeing overall reductions in antimicrobial use, the interrupted time series showed an immediate effect on overall antimicrobial use but did not show a sustained decrease even with the adjustment of the 3-month lag to account for slow uptake of intervention acceptance. This is possibly due to the interrupted time series analysis capturing the month-to-month variation in prescribing, instead of averaging antimicrobial use over the entire pre- and post-intervention periods. However, when performing the interrupted time series on the ASI, there was both an immediate and sustained decrease. This is likely related to the significant reduction in use of certain broad-spectrum agents like fluoroquinolones and cefepime. The ASI was designed to better quantify the impact of antimicrobial stewardship programs on the spectrum of antimicrobial agents used especially for interventions related to de-escalation of therapy, which was the second most common intervention type in our study. Other studies have shown that while an intervention may not impact overall antibiotic usage, metrics assessing antimicrobial spectrum may reveal prescribing trends resulting in the selection of more narrow-spectrum agents.^15^ Our handshake stewardship intervention was able to affect both usage and spectrum.

In this study, there were no differences found in patient outcomes including in-hospital mortality, ICU length of stay, 30-day readmission, or hospital-onset *C. difficile* infection. However, hospital length of stay significantly increased during the post-implementation period. It is unclear if this finding was influenced by unaccounted-for differences in baseline characteristics, surgical procedure types/complexity, and/or severity of illness. It does not appear that the increase in hospital length of stay is related to increased infectious complications as there was no change in 30-day readmission due to any cause including re-infection related to less or narrower spectrum antibiotics. The increased length of stay is more likely a reflection of the nationally experienced trend in overall increased lengths of stay for all hospitalizations following the COVID-19 pandemic.^8,16^

Limitations in this retrospective study are mostly related to tracking capabilities. In this analysis, only inpatient DOTs were included; therefore, it is unclear whether handshake antimicrobial stewardship resulted in less post-discharge antibiotic use. Antimicrobial indication or use for empiric, definitive treatment, or prophylaxis was not included in this analysis. This study did not assess whether there was an impact on appropriateness of antibiotic use. Whether or not the statistically significant differences in DOT were clinically meaningful was also not able to be fully elucidated. Lastly, there is a possibility that the data collected and analyzed is missing confounders that may have affected length of stay.

In conclusion, handshake antimicrobial stewardship was successfully expanded to include adult general surgical units and resulted in a shift towards narrower antibiotic ordering and an overall reduction in antibiotic usage.

## Supporting information

Kosharek et al. supplementary material Kosharek et al. supplementary material

## Supplementary material

For supplementary material accompanying this paper visit http://doi.org/10.1017/ash.2024.498. click here to view supplementary material
