# Supplementary material for: Handshake antimicrobial stewardship for adult surgical patients
Source: Antimicrob Steward Healthc Epidemiol. 2025 Feb 12;5(1):e46. doi: 10.1017/ash.2024.498 (PMC11822574; doi:10.1017/ash.2024.498)
Supplement: Kosharek et al. supplementary material [file S2732494X24004984sup001.docx]

Supplementary Appendix

Supplemental Methods

Table 1. Existing Antimicrobial Stewardship Program Details

| ASP Strategy | Description |
| --- | --- |
| Prior Authorization | Antimicrobials included: Amphotericin B, liposomal, Artesunate, Cabotegravir-Rilpivirine, Cefiderocol, Ceftazidime-avibactam, Ceftolozane-tazobactam, Dalbavancin, Eravacycline, Imipenem-relebactam, Meropenem-vaborbactam, Tedizolid |
| 72 hour Prospective audit and feedback | Antimicrobials included: Ceftaroline, Ceftazidime, Colistin, Cidofovir, Daptomycin, Foscarnet, Maribavir, Quinupristin-dalfopristin, inhaled Ribavirin, Telavancin, Tigecycline  Blood cultures with rapid diagnostic results |
| Restricted by Use Criteria | Antimicrobials included: Aztreonam, Baloxavir, Letermovir, Remdesivir, SUBA-itraconazole |
| Guidelines and order sets | C. difficile Treatment Guidelines  COVID-19 Treatment Guidelines  Intra-abdominal Infection Guidelines  Pneumonia Treatment Guidelines  BioFire Pneumonia Panel Treatment Guidelines  Sexually Transmitted Infection Guidelines  Skin and Soft Tissue Infection Guidelines  Surgical Antimicrobial Prophylaxis Guidelines  Urinary Tract Infection for Hospitalized Patients Guidelines |
| Renal dosing policy | No automatic pharmacist renal dosing policy. However we utilize renal dose context on order entry to guide prescribers to the initial correct renal dose. In addition, we have retrospective renal dosing clinical decision support rules that alert pharmacists 365 days/year when the dose ordered does not match the recommended dose. |
| Automatic IV to PO policy | There was a pharmacist Medication Therapy Management protocol for IV to PO antimicrobials in existence until December 2021 but given the low utilization of pharmacists using this automatic protocol (average 3 per month) compared to the number of IV to PO interventions being made and accepted outside of the confines of the protocol (average 55 per month) it was retired. |
| Clinical decision support alerts | These alerts are used to create a scoring system that are reviewed by pharmacists daily to identify patients that may need an ASP-related intervention. These include insufficient therapy alerts, de-escalation alerts, drug resistance alerts, drug lab alerts, and duplicate therapy alerts. |
| Allergy related initiatives | In March 2021 the penicillin-cephalosporin cross reactivity notification was modified to alert prescribers upon order entry only in patients with severe (e.g. anaphylaxis) penicillin allergies. |
| Antibiotic Time Outs | In March 2022 the electronic antimicrobial time out that fired at 72 hours for anti-MRSA and anti-Pseudomonal antibiotics was removed from the electronic health record. This was based on internal data showing providers acted on this alert < 50% of the times it fired. An internal review demonstrated no increase in anti-MRSA and anti-Pseudomonal use in the 1 year post de-implementation. |

Additional details regarding handshake ASP rounds

- Prior to expanding to the surgical floor services, email communication was sent from ASP to surgical leadership describing the service and rationale including positive experience on the medicine floor services.
- The two general surgical floors (61 beds) housed only patients from one of the three general surgery teams – trauma, geriatric trauma, or acute and critical care surgery teams. Any patient with trauma, geriatric trauma, or acute and critical care surgery teams that was not housed on one of these two surgical floors was not included in the analysis. Patients in the surgical intensive care unit were not included in the intervention or analysis.
- Pre-round review of all patients receiving antimicrobials split between an ID physician (1 FTE) and ID pharmacist (1 FTE), approximately 40 patients per day, ~1 hour
  - Same two individuals performed the majority (>90%) of handshake interventions. Additional support from other ID physicians and pharmacy residents occurred in minority of interventions.
  - Patients were identified from a report in the EHR identifying all patients receiving systemic antimicrobials. Patients were then filtered by location and primary service. Patients with formal ID consults were excluded from review by the handshake ASP team.
- Sit-down rounds to discuss potential interventions, ~1 hour
- In person rounds to discuss recommendations with the general surgical team physician associates or nurse practitioners responsible for floor management of surgical patients, ~1 hour
  - Rounds typically occurred on Wednesdays around 11:00 but this was not scheduled
  - The ASP team would visit the primary team workroom on the surgical floors
- Post-rounds intervention documentation, ~30 minutes
  - Interventions were input by the Handshake antimicrobial stewardship team after rounds using the institutional Epic Antimicrobial Stewardship Standard Operation Procedure. Definitions of Antimicrobial Stewardship Intervention types:
    - Broaden coverage/address absent therapy: Use when empiric or pathogen-recommended antimicrobial therapy is recommended to be broadened due to insufficient coverage.
    - De-escalation: Use when antimicrobial therapy has been de-escalated either empirically or based on culture results.
    - Discontinue: Use when an antimicrobial is discontinued and not replaced with another.
    - Dose optimization: Use when an antimicrobial dose and/or frequency is changed.
    - Lengthen Duration: Use when a duration of therapy is extended.
    - Medication safety: Use when a patient’s antimicrobial is switched to an alternative safer regimen due to drug interaction or contraindication.
    - Monitoring-related: Use when recommending a non-therapeutic drug level lab (ex. Requesting additional susceptibilities from microbiology).
    - Route change intravenous to oral: Use when changing the same antibiotic from intravenous to oral or per tube.
    - Shorten/define duration: Use when a patient’s duration of therapy of shortened or a previous unclear duration of therapy is clarified.

Interventions were reviewed for acceptance by the ID pharmacist the next business day and any recommendations that were implemented within 24 hours were counted as accepted.

Additional method details

- Charlson Comorbidity Index was collected electronically using ICD-10 codes
- Charlson Comorbidity Index reported was the age-adjusted score

Modified Antimicrobial Spectrum Index

Changes from the original Antimicrobial Spectrum Index as defined by Gerber JS, et al. Highlighted in bold.

Gerber JS, Hersh AL, Kronman MP, Newland JG, Ross RK, Metjian TA. Development and Application of an Antibiotic Spectrum Index for Benchmarking Antibiotic Selection Patterns Across Hospitals. Infect Control Hosp Epidemiol. 2017 Aug;38(8):993-997. doi: 10.1017/ice.2017.94. Epub 2017 May 31. PMID: 28560946.

Supplemental Results

Table 2. Additional characteristics of primary procedure performed during admission

| **Characteristics of Primary procedure** | **Pre-Implementation**  Jul 2020 – Dec 2021  (N = 1,822) | **Post-Implementation**  Jan 2022 – June 2023  (N = 1,769) |
| --- | --- | --- |
| American Society of Anesthesiologists (ASA) classification, n (%)  1  2  3  4  5  Not reported | 76 (4%)  562 (31%)  905 (50%)  208 (11%)  35 (2%)  36 (2%) | 41 (2%)  469 (26.5%)  999 (56.5%)  176 (10%)  19 (1%)  65 (4%) |
| Primary procedure description simplified, n (%)  Amputation or amputation revision  Appendectomy or laparoscopic appendectomy  Application of external fixation  Arthroplasty or revision arthroplasty  Cholecystectomy or laparoscopic cholecystectomy  Debridement  Esophagogastroduodenoscopy*  Endoscopic retrograde cholangiopancreatography**  Excision hidradenitis/cyst/lesion/mass  Exploration laparotomy  Exploration wound  Fusion spinal (cervical, thoracic, or lumbar)/Laminectomy  Gastrostomy tube insertion  Hemiarthroplasty  Hernia repair^  Incision and drainage  Intramedullary nailing  Laparoscopic other#  Open reduction internal fixation  Other  Other cardiovascular/cardiothoracic  Other Neurosurgery  Other Ophthalmology  Other Orthopedics  Other Otolaryngology  Other Plastics  Other Vascular  Percutaneous sacral-iliac screw placement or removal/exchange | 49 (2.7%)  47 (2.6%)  32 (1.8%)  32 (1.8%)  40 (2.2%)  136 (7.5%)  25 (1.4%)  23 (1.3%)  11 (0.6%)  229 (12.6%)  31 (1.7%)  56 (3.1%)  12 (0.7%)  65 (3.6%)  33 (1.8%)  189 (10.4%)  203 (11.1%)  19 (1.0%)  313 (17.2%)  93 (5.1%)  10 (0.5%)  10 (0.5%)  16 (0.9%)  65 (3.6%)  10 (0.5%)  8 (0.4%)  45 (2.5%)  20 (1.1%) | 58 (3.3%)  46 (2.6%)  18 (1.0%)  44 (2.5%)  54 (3.1%  124 (7.0%)  20 (1.1%)  12 (0.7%)  8 (0.5%)  187 (10.6%)  16 (0.9%)  64 (3.6%)  10 (0.6%)  83 (4.7%)  33 (1.9%)  166 (9.4%)  256 (14.5%)  24 (1.4%)  288 (16.3%)  79 (4.4%)  9 (0.5%)  8 (0.5%)  20 (1.1%)  63 (3.6%)  27 (1.5%)  12 (0.75)  20 (1.1%)  20 (1.1%) |

*Including with biopsy, stent placement, endoscopic ultrasound, band ligation, removal foreign body, drain pseudocyst, control bleeding, place percutaneous endoscopic gastrostomy, balloon dilation, place catheter/tube, ultrasound fine needle aspiration/biopsy

**Including with stent placement, remove/exchange stent, removal stones

^Including incisional, inguinal, umbilical, ventral with and without mesh

#Including diagnostic, hemicolectomy, gastric bypass, splenectomy, insertion gastrostomy tube

Figure 1.


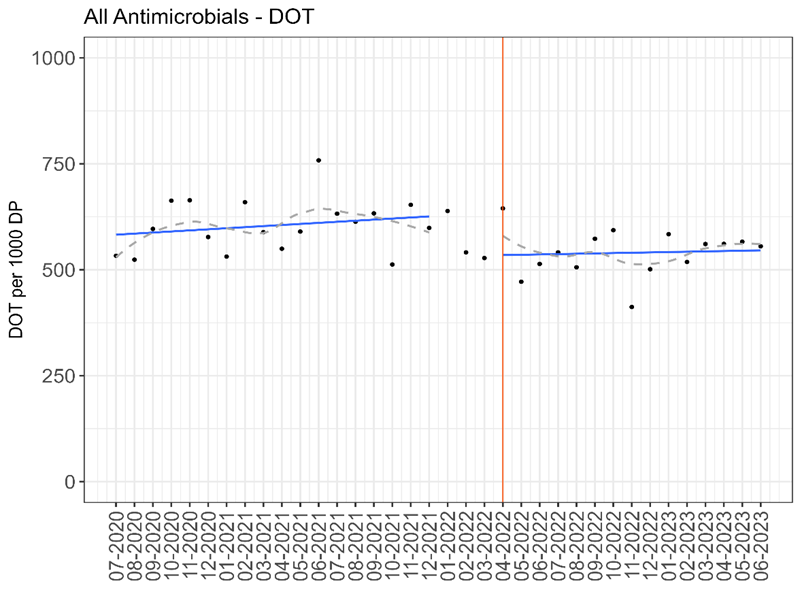


Antimicrobial usage by month on general surgery units (black dots); average consumption from the predicted distribution of the interrupted time series segmented regression model (blue line) with loess smoother (gray dashed line); red line indicates 3-month lag from intervention start.

Figure 2.


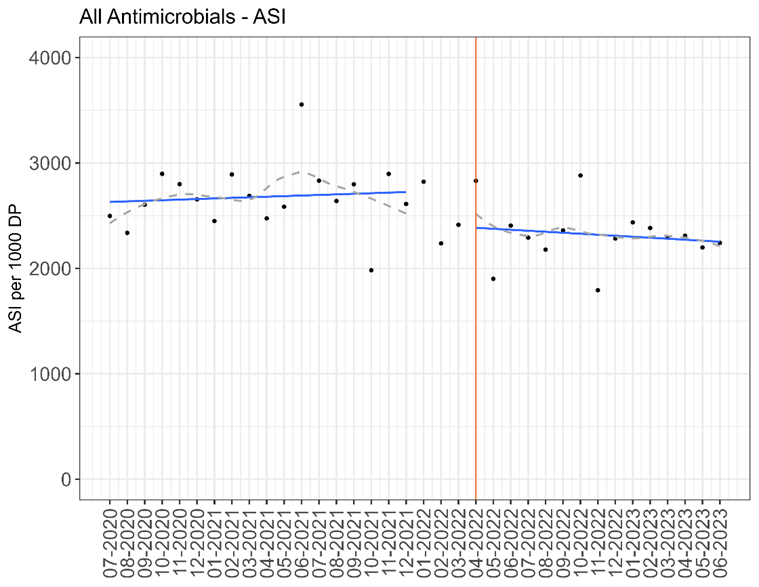


Antimicrobial usage by month on general surgery units (black dots); average spectrum score from the predicted distribution of the interrupted time series segmented regression model (blue line) with loess smoother (gray dashed line); red line indicates 3-month lag from intervention start.

Figure 3.


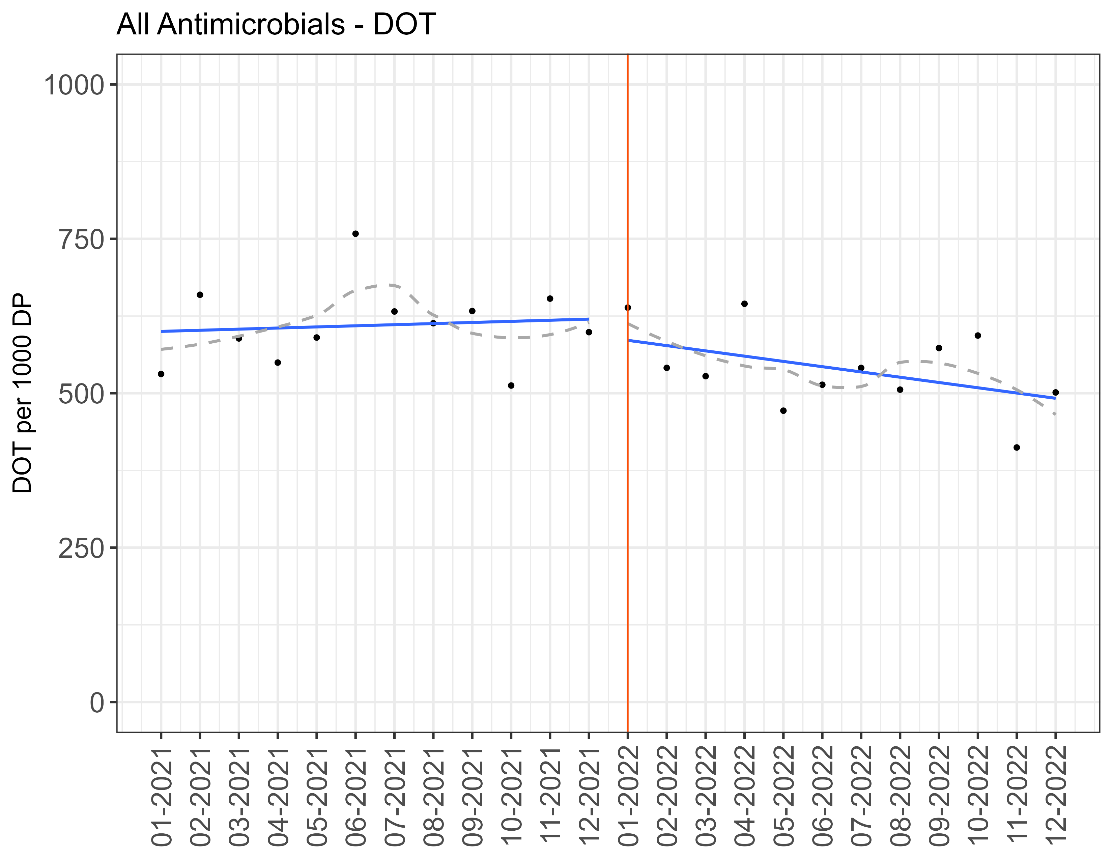


Additional analysis requested by reviewers looking only at a 12-month pre-intervention interval. Antimicrobial usage by month on general surgery units (black dots); average consumption from the predicted distribution of the interrupted time series segmented regression model (blue line) with loess smoother (gray dashed line); red line indicates intervention start.
